# Evidence for a reproductive sharing continuum in cooperatively breeding mammals and birds: consequences for comparative research

**DOI:** 10.1098/rspb.2023.0607

**Published:** 2023-09-13

**Authors:** Yitzchak Ben Mocha, Tal Dahan, Yuqi Zou, Michael Griesser, Shai Markman

**Affiliations:** ^1^ Department of Evolutionary and Environmental Biology, University of Haifa, 3498838 Haifa, Israel; ^2^ Department of Biology and Environment, University of Haifa at Oranim, 36006 Tivon, Israel; ^3^ Department of Biology, University of Konstanz, Universitätsstrasse 10, 78457 Konstanz, Germany; ^4^ Center for the Advanced Study of Collective Behavior, University of Konstanz, Universitätsstrasse 10, 78457 Konstanz, Germany; ^5^ Department of Collective Behaviour, Max Planck Institute of Animal Behaviour, Universitätsstrasse 10, 78457 Konstanz, Germany

**Keywords:** communal breeding, cooperative breeding, mammals, birds, maternity, reproductive skew

## Abstract

Extreme reproductive skew occurs when a dominant female/male almost monopolizes reproduction within a group of multiple sexually mature females/males, respectively. It is sometimes considered an additional, restrictive criterion to define cooperative breeding. However, datasets that use this restrictive definition to classify species as cooperative breeders systematically overestimate reproductive skew by including groups in which reproduction cannot be shared by definition (e.g. groups with a single female/male). Here, we review the extent of reproductive sharing in 41 mammal and 37 bird species previously classified as exhibiting alloparental care and extreme reproductive skew, while only considering multi-female or multi-male groups. We demonstrate that in groups where unequal reproduction sharing is possible, extreme reproductive skew occurs in a few species only (11/41 mammal species and 12/37 bird species). These results call for significant changes in datasets that classify species' caring and mating system. To facilitate these changes, we provide an updated dataset on reproductive sharing in 63 cooperatively breeding species. At the conceptual level, our findings suggest that reproductive skew should not be a defining criterion of cooperative breeding and support the definition of cooperative breeding as a care system in which alloparents provide systematic care to other group members’ offspring.

## 1. Introduction

Cooperative breeding is a care system with the core element of conspecifics providing parental care to the offspring of other group members (i.e. alloparental care) [1,2]. For instance, in a range of species, alloparents provide systematic allonursing [3,4], allofeeding [5,6], babysitting [3,7] and transference of offspring between locations [8,9]. In some cases, alloparents even care for others' offspring while not reproducing themselves, leading to reproductive skew within social groups (e.g. common mole-rat, *Cryptomys hottentotus hottentotus* [10]; Florida scrub jay, *Aphelocoma coerulescens* [11]).

Within-group reproductive skew is of fundamental importance in cooperative-breeding research because it is sometimes considered a defining criterion of cooperative breeding (especially in mammalogy, reviewed in [12,13]). For example, some defined cooperative breeding in mammals as a social system where ‘juveniles are cared for by their parents and by non-breeding helpers of either or of both sexes’ [14]. Similarly, others defined cooperative breeding in birds as a breeding system in which helpers do not breed or have ‘zero-to-limited opportunities to breed’ [15]. This restrictive approach to define cooperative breeding therefore classifies species that engage in alloparental care but share within-group reproduction as communal breeders, rather than cooperative breeders [14,16].

Comparative studies that use such restrictive definitions of cooperative breeding are thus critically dependent on the correct identification of species exhibiting both definitional criteria of cooperative breeding: alloparental care and extreme female and/or male reproductive skew [12,17]. Nonetheless, datasets that use this restrictive definition to classify species' social systems have two crucial shortcomings. First, they systematically overestimate the prevalence of extreme reproductive skew across species. Second, they do not indicate species whose classification of the caring system is based on poor data, thereby preventing researchers from accounting for uncertainty in the classification. Below, we discuss these shortcomings and their consequences for comparative research of cooperative breeding.

### (a) Shortcoming I: overestimation of reproductive skew

Ideally, within-group reproductive skew should be calculated using an index controlling for all factors affecting unequal reproductive distribution including, for instance, group size, age and kinship [18,19]. Clearly, such indices require detailed longitudinal data [18,20] that are rarely available for wild animal populations. To overcome this deficiency of data and increase the sample size of species, comparative studies often use rough proxies of reproductive skew; for instance, the percentage of offspring born to the most dominant females in social groups [21,22]. Critically, these percentages are frequently calculated from the reproductive output of all sampled groups, including those with a single sexually mature female/male (hereafter ‘single-female groups’ or ‘single-male groups’). This approach, however, contradicts the fundamental rationale of determining reproductive skew: i.e. measuring the deviation from equal reproductive distribution within a group of multiple *potentially* reproductive individuals of a specific sex [16,18,20,23–25]. Measuring reproductive ‘skew’ or ‘suppression’ is only meaningful where the reproductive potential of at least one group member can be compromised (i.e. group size of ≥2 sexually mature individuals of the sex in question; hereafter ‘multi-female groups’ or ‘multi-male groups’) [16,26–28].

### (b) Shortcoming II: controlling for the certainty of species classification

A second important shortcoming is that, despite the paucity of parentage data in many species, current datasets of mammal and bird species often do not indicate species whose mating or caring systems are classified based on limited sample size or potentially biased data (e.g. data from experimental populations; see also [12,17]). Consequently, comparative studies that use these datasets have to treat the classification of all species with the same degree of confidence, although the availability of statistical methods to account for data uncertainty.

### (c) Revisiting the underlying biological assumption of the restrictive definition of cooperative breeding

**Figure 1.**
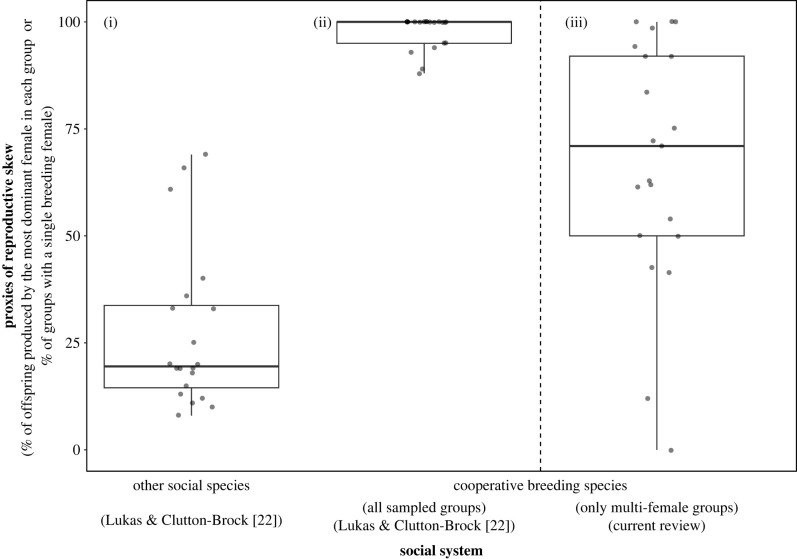
Proxies of female reproductive skew in social mammals (amended from Lukas & Clutton-Brock [22]). The reproductive output of dominant females in (*i*) 18 ‘other’ social species as reported by Lukas & Clutton-Brock [22] (note that although Lukas & Clutton-Brock report a sample size of 20 species, the dataset in the electronic supplementary materials includes only 18 unique species), (*ii*) 21 cooperative breeding species as reported by Lukas & Clutton-Brock [22], and (*iii*) the same 21 species as in (*ii*) while we only considered multi-female groups (see electronic supplementary material, table S1 for data). Horizontal bars represent the median, boxes the 25% and 75% quartile and vertical lines indicate the minimum and maximum values. Notes: (i) ‘other social species’ combines ‘communal’ breeding species (i.e. species where ‘most adult females breed regularly and share care’, e.g. banded mongoose) and other social species (i.e. species where ‘females live in groups and are neither cooperative nor communal breeders’, e.g. chimpanzee [22]). In (iii), for species with multiple data points (e.g. from different populations), all data points from samples containing only multi-female groups were used to calculate a mean weighted by the sample size of each data point. Note, that in the absence of samples containing only multi-female groups, we considered mixed samples for five species (see electronic supplementary material, table S1 for details). We excluded another five species that were classified as cooperative breeders by Lukas & Clutton-Brock [22] from (ii) and (iii) because we could not find data on reproductive sharing (Eurasian beaver, *Castor fibre*; Ochre mole-rat, *Cryptomys ochraceocinereus*; side-striped jackal, *Canis aureus*; see electronic supplementary material, table S2 for current taxonomy) or confirm alloparental care in these species (California mouse, *Peromyscus californicus*; Oldfield mouse, *Peromyscus polionotus*; see electronic supplementary material, table S2 for details).

Given these two shortcomings, we revisited the biological assumption underlying the restrictive definition of cooperative breeding. Namely, the biological assumption that there is a quantitative distinction between the reproductive skew of two types of species with alloparental care [16,22]: species with alloparental care and extreme within-group reproductive skew (i.e. cooperative breeders) and species with alloparental care and low within-group reproductive skew (i.e. communal breeders). This distinction between cooperative and communal breeding species was discussed in theoretical papers [14,29,30] and was used to classify species in numerous comparative studies [15,16,31,32]. Nonetheless, to the best of our knowledge, its only empirical support comes from a comparative study on mammals, claiming that dominant females in cooperative breeding species produce 88–100% of offspring, while in other social mammals (with and without alloparental care) dominant females produce 8–69% of offspring [22] (figure 1). However, if the assumption about a quantitative gap between the reproductive skew in cooperative and communal breeding species is incorrect, we predict finding a continuum of reproductive sharing among species with alloparental care [33,34].

To test this prediction, we re-estimated the degree of reproductive sharing in the 41 mammal and 37 bird species that were classified as exhibiting alloparental care and extreme female and/or male reproductive skew in at least one of four frequently used datasets: Lukas & Clutton-Brock [22], Federico *et al*. [16], Raihani & Clutton-Brock [21] and Cornwallis *et al.* [15]. To avoid overestimation of reproductive skew, we only considered social groups with multiple sexually mature females and/or males, and avoided other biases that may cause an overestimation of reproductive skew when data permitted (see §2c). To facilitate future research, we provide estimations for the certainty of the classification of each species as exhibiting extreme reproductive skew or not (see §2e).

## 2. Methods

### (a) Revisited datasets

In mammals, we revisited the classification of 41 species that were classified as exhibiting alloparental care and an extreme female reproductive skew in at least one of three datasets: Raihani & Clutton-Brock [21] (seven species classified as exhibiting alloparental care and more than 90% of reproductive output belongs to the dominant female in the group), Lukas & Clutton-Brock [22] (34 species classified as exhibiting alloparental care and more than 90% of reproductive output belongs to the dominant female in the group) and/or Federico *et al*. [16] (13 species classified as exhibiting alloparental care and ‘only dominant individuals breed’, p. 1038). Following the curators of these datasets, we only examined the female reproductive skew for mammals.

In birds, we revisited the classification of 37 species that were classified as exhibiting alloparental care and extreme female reproductive skew in at least one of two datasets: Raihani & Clutton-Brock [21] (24 species classified as exhibiting alloparental care and more than 90% of reproductive output belongs to the dominant female in the group) and/or Cornwallis *et al.* [15] (36 species classified as exhibiting alloparental care and helpers that do ‘not breed or had zero-to-limited opportunities to breed’). As Cornwallis *et al.* did not indicate the sex of helpers, for birds, we examined reproductive skew among females and males. Yet, the common eider (*Somateria mollissima*) was excluded from the male dataset as it was only classified as a cooperative breeder by Raihani & Clutton-Brock [21] who examined female reproductive skew.

### (b) Literature review

For each of these 41 mammal and 37 bird species, we searched data on reproductive sharing via three steps. First, we reviewed the citations that were used to support the species' classification in the datasets that classified this species. Second, if this reference indeed included relevant data on parentage, we used Google Scholar to identify studies that cited this supporting reference. The title and/or abstract of each paper found in this search were reviewed and relevant papers were examined further. Third, to ensure a comprehensive review of the literature on each species, we searched Web of Science using the following search command (TOPIC = English species name* AND (extra pair* OR extrapair* OR extra-pair* OR cooperative breeding* OR alloparental care* OR helpers* OR social system* OR parentage* OR maternity* OR paternity* OR genetic* OR DNA* OR reproduction*)) OR (TOPIC = Latin species name * AND (extra pair* OR extrapair* OR extra-pair* OR cooperative breeding* OR alloparental care* OR helpers* OR social system* OR parentage* OR maternity* OR paternity* OR genetic* OR DNA* OR reproduction*)). The title and/or abstract of each paper found in these searches were reviewed and relevant papers were examined. This procedure was repeated until no more new papers were found. Our review was limited to publications in English.

In addition, we revisited the occurrence of alloparental care in each of the examined species. Since the focus of this study is the prevalence of extreme reproductive skew, we used a relaxed definition and considered any type of care provided by non-parents as alloparental care [12,35]. Note, that some scholars may reject certain behaviours as alloparental care (e.g. thermoregulation in Alpine marmots [36]) and, consequently, also the classification of these species as cooperative breeders (see discussions in [12,35]).

Only primary literature describing wild populations was considered relevant for our review. We did not consider secondary literature, including reviews and data from experimental groups, captive groups or introduced populations [37]. As the species were reviewed from 2021 to 2023, our results reflect the literature published before 2021.

### (c) Proxies of reproductive skew

We calculated two proxies of reproductive skew. First, we summed the number of offspring produced by the most dominant female/male across all groups with multiple sexually mature females/males and calculated the percentage of this number out of the total number of offspring produced in these groups (hereafter the female/male ‘parentage-skew’ proxy, respectively).

Second, we calculated the percentage of groups that had a single breeding female/male out of the total number of groups with multiple sexually mature females/males (hereafter the female/male ‘skew-of-groups’ proxy, respectively). As a rule, we calculated the skew-of-groups proxy out of the total number of years during which the groups were sampled (i.e. including multiple samples of the same social group over several breeding attempts; hereafter ‘group-years’). However, in groups where the composition of adults remained the same across years, we used the number of groups (and not group-years) to calculate the skew-of-groups proxy (e.g. apostlebird, *Struthidea cinerea*). This approach enables to detect reproductive sharing that occurs over multiple breeding attempts [37–40], and it is particularly important in species with a small clutch size where reproduction cannot be shared within the same breeding attempt (e.g. Seychelles warblers, *Acrocephalus sechellensis*, in which 67% of clutches have one egg [41]).

**Figure 2.**
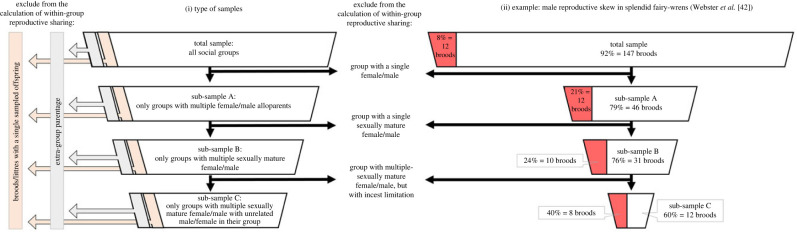
The effect of social composition on the estimation of within-group reproductive sharing. (*i*) The upper box represents the total sample including all social groups. Each lower box is a sub-sample of the sample above it after excluding groups with the social composition indicated to the right. To focus only on within-group reproductive sharing, extra-group parentage should be excluded from all the samples. In studies that sample each social group once, broods/littres with a single sampled offspring should be excluded from all samples because shared parentage cannot be detected/occur in these groups. (*ii*) An example demonstrating how the social composition of the sample affects the estimation of within-group male reproductive sharing in splendid fairy-wrens (*Malurus splendens*; based on data from Webster *et al.* [42], pp. 912–913). Each box represents a sample of broods. The red/white areas within each box represent the proportion of broods in which male alloparents did/did not sire offspring in their group, respectively. Using the threshold of more than 90% broods with single paternity of the dominant male, splendid fairy-wrens will be classified as exhibiting extreme male reproductive skew only in the total sample (note that in this species, 64% of groups consist of a pair with no alloparents and that 20% of broods entirely sired by a male other than the dominant male in the group). Sub-sample B consists only of the broods from multi-male groups for which the genetic relationship between the alloparent male and the breeding female was known. The number of broods with a single sampled offspring and the number of broods consisting only of extra group parentage were not indicated in Webster *et al.* [42] and could not, therefore, be excluded from this example.

Where data were detailed enough, the following parentage data were also excluded from the calculation of reproductive skew proxies (figure 2; see electronic supplementary material, table S1 for details for each sample):

(i) Groups without at least one adult male and female (since they are not functionally reproductive units).
(ii) Extra-group parentage was excluded since restrictive definitions of cooperative breeding concern within-group reproductive sharing [43]. Specifically, offspring whose mother/father were animals outside of the focal group were excluded from the maternity/paternity parentage-skew proxies. Broods in which all eggs were laid/fertilized by animals outside of the social group were excluded from the female/male skew-of-groups proxies [37].
(iii) Where the skew-of-groups proxy was not calculated across multiple breeding attempts of the same group (see above), clutches/litters with only one sampled offspring were excluded (since shared maternity/paternity cannot be detected/occur in these clutches/litters [26]).
(iv) Groups in which only one female/male was unrelated to the opposite sex breeder (i.e. groups with incest limitation on alloparents [44,45]).

We calculated reproductive skew proxies from each study that included relevant data and represented different populations, study periods or types of samples (e.g. samples of groups in which alloparents were/were not related to the opposite-sex breeder). Hence, most species have multiple data points that represent this within-species variation [37].

### (d) Binary classification of species exhibiting extreme reproductive skew or not

We classified whether each species exhibits a female (for mammals and birds) or male (for birds only) extreme reproductive skew (yes/no). As a conservative approach, we used the more relaxed threshold used by curators of the examined datasets [21,22] and others [45]: a species was considered to exhibit an extreme female/male reproductive skew if the most dominant female/male in each group produced more than 90% of offspring (i.e. the parentage-skew proxy) or if more than 90% of the groups had a single breeding female/male (i.e. the skew-of-groups proxy).

What constitutes the social group from which proxies of reproductive skew should be calculated differ significantly between species [45]. For each species, we considered the basic social unit as regarded by the authors of the papers examined for parentage. For most species examined, the basic social unit consisted of two or more breeders with or without associate alloparents (e.g. Arabian babblers: *Argya squamiceps*, meerkats: *Suricata suricatta*). However, in species living in multi-level societies, the basic social unit may consist of several reproductive sub-units [46]. Species living in multi-level societies where the basic social unit included more than one breeding pair were therefore considered to not exhibit extreme reproductive skew even if breeding pairs were genetically monogamous (white-fronted bee-eater, *Merops bullockoides*; superb starling, *Lamprotornis superbus*; bell miner, *Manorina melanophrys*; see electronic supplementary material, table S2 for details). Note that our classification of these species as ‘plural breeders’ is in accordance with the primary literature on reproductive sharing in these species (white-fronted bee-eater [47], superb starling [48], bell miner [49]). For example, the basic social unit of white-fronted bee-eaters is a ‘clan’ consisting of an extended family with up to four overlapping generations that often include multiple breeding pairs [50,51]. Birds that do not breed at the current breeding season and failed breeders provide alloparental care to other pairs within their clan [51]. Thus, although breeding pairs are genetically monogamous [50], we considered the species as a plural breeder due to the presence of multiple breeding pairs within the basic social unit.

#### (i) Species with ambiguous proxies

**Figure 3.**
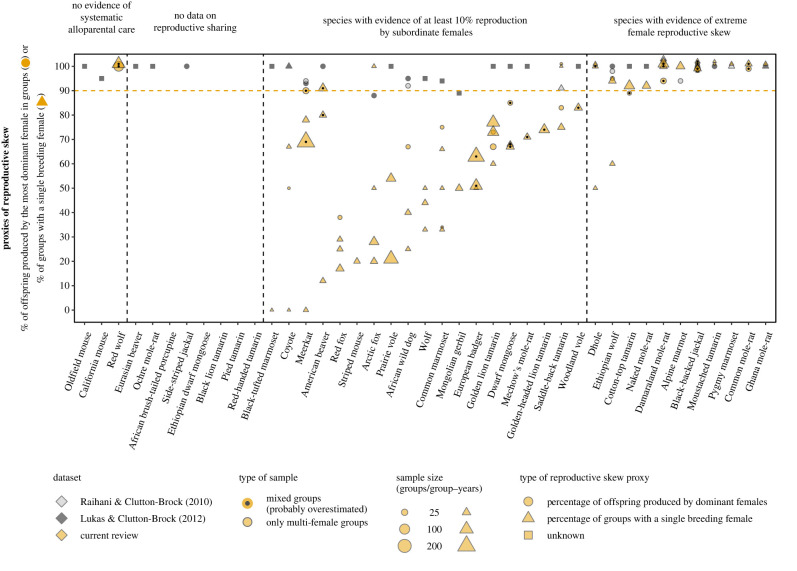
Within-group female reproductive sharing in the 41 mammal species classified as exhibiting alloparental care and an extreme female reproductive skew in Raihani & Clutton-Brock [21] and/or Lukas & Clutton-Brock [22] and/or Federico *et al*. [16] datasets. The horizontal line represents Raihani & Clutton-Brock's [21] minimum threshold for extreme female reproductive skew. Mixed samples may include groups with a single sexually mature female and therefore likely overestimate reproductive skew. Multiple data points for the same species represent different sample types, study periods or populations. Data from Raihani & Clutton-Brock [21] and Lukas & Clutton-Brock [22] are not adjusted for sample size as these reviews did not report sample size for most species. Species without data points are species for which we could not find data and/or the examined datasets did not include quantitative values to support their classification. For clarity, overlapping data points for the same species were separated by placing one of them one percentage higher.

**Figure 4.**
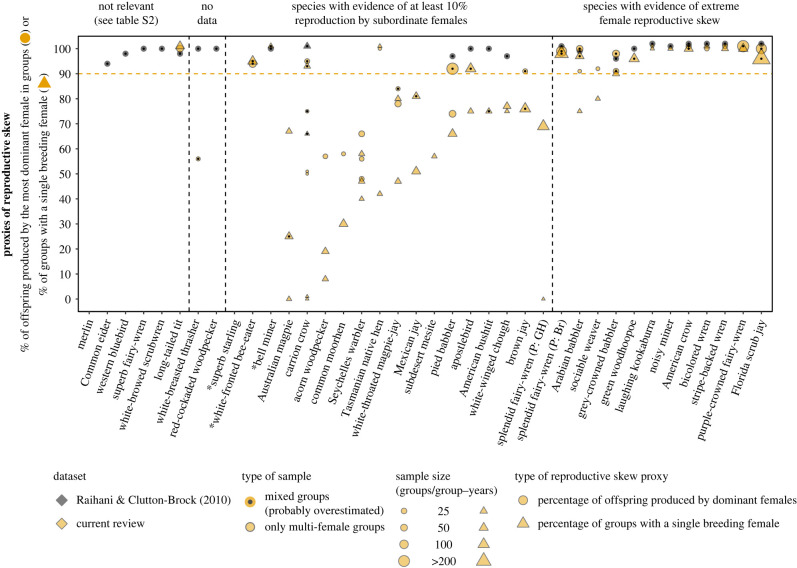
Within-group female reproductive sharing in the 37 bird species classified as exhibiting alloparental care and an extreme female reproductive skew in Cornwallis *et al.* [15] and/or Raihani & Clutton-Brock [21]. The horizontal line represents Raihani & Clutton-Brock's [21] minimum threshold for extreme female reproductive skew. Mixed samples may include groups with a single sexually mature female and, thus, likely overestimate reproductive skew. Multiple data points for the same species represent different samples, study periods or populations. Data from Raihani & Clutton-Brock [21] are not adjusted for sample size. Species without data points are species for which we could not find data and/or the examined datasets did not provide quantitative values to support their classification. For clarity, overlapping data points for the same species were separated by placing one of them one percentage higher. Species with an asterisk live in multi-level societies in which the basic social unit consists of multiple breeding pairs and these species were, therefore, considered plural breeders (see electronic supplementary material, table S2 for details). Splendid fairy-wren is represented twice as two populations of the species exhibit different degrees of reproductive skew (see electronic supplementary material, tables S1 and S2 for details).

**Figure 5.**
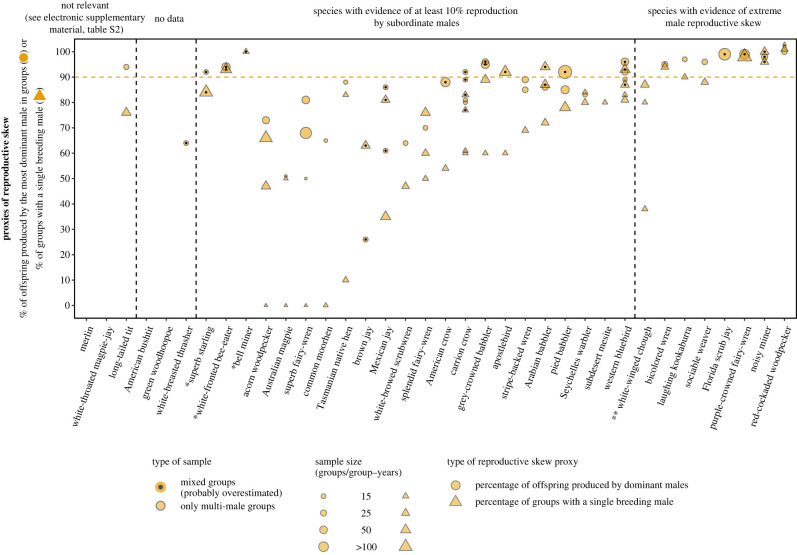
Within-group male reproductive sharing in the 36 bird species classified as exhibiting alloparental care and an extreme reproductive skew in Cornwallis *et al.* [15]. The horizontal line represents Raihani & Clutton-Brock's [21] minimum threshold for extreme reproductive skew. Mixed samples may include groups with a single sexually mature male and, thus, likely overestimate reproductive skew. Multiple data points for the same species represent different samples, study periods and/or populations. Species without data points are species for which we could not find data. For clarity, overlapping data points for the same species were separated by placing one of them one percentage higher. Species with an asterisk live in multi-level societies in which the basic social unit consists of multiple breeding pairs and these species were, therefore, considered plural breeders (see electronic supplementary material, table S2 for details). White-winged chough is marked with two asterisks because reproduction is shared in newly formed groups, but it is usually monopolized in socially established groups (see electronic supplementary material, table S2 for details).

For six mammal species (American beaver, *Castor canadensis*; Arctic fox, *Vulpes lagopus*; cotton-top tamarin, *Saguinus oedipus*; dhole, *Cuon alpinus*; Ethiopian wolf, *Canis simensis*; saddle-back tamarin, *Saguinus fuscicollis*) and nine bird species (apostlebird*;* Arabian babbler*;* brown jay, *Cyanocorax morio*; grey-crowned babbler, *Pomatostomus temporalis*; laughing kookaburra, *Dacelo novaeguineae*; sociable weaver, *Philetairus socius*; splendid fairy-wren; Tasmanian native hen, *Tribonyx mortierii*; white-winged chough, *Corcorax melanorhamphos*), we found multiple proxies, some were below and others above the threshold of extreme reproductive skew (e.g. due to different sample types, periods or locations). We report all these proxies in figures 3–5 and the electronic supplementary material, table S1. Yet, for the binary classification of these species (exhibiting extreme reproductive skew or not), we prioritized data as follows (see details per species in electronic supplementary material, table S2):

(i) Data from a sample that included only multi-female groups/multi-male groups override data from a mixed sample of single and multi-female/multi-male groups (hereafter ‘mixed sample’). Data from mixed samples were only considered if a sample including solely multi-female or multi-male groups was small (i.e. less than 15 group-years), represented a different population or was not available (though mixed samples are presented in figures 3–5 for comparison). When dominance ranks of group members were known for both proxies, data for the parentage-skew proxy override data for the skew-of-groups proxy.
(ii) Genetic evidence overrides behavioural or physiological evidence of parentage (e.g. lactation, the number of placental scars or abnormally large clutch/litter size).
(iii) Studies with larger sample sizes override studies with smaller sample sizes.
(iv) Reproductive suppression may occur at any reproductive stage from interference with copulation [52,53] until infanticide [54,55]. We therefore prioritized data on the reproductive output in later reproductive stages as they are less likely to be aborted in the following order: number of weaned/fledged offspring, number of dependent offspring and number of pregnant females. For a valid comparison between dominant and subordinate group members, per study, we only considered the type of reproductive output that was available for both dominance categories.

### (e) Evaluation of the certainty of species classification

We evaluated the strength of evidence for each species' classification qualitatively and quantitatively. Qualitatively, we indicated the type of sample used in each study (i.e. samples consisting only of multi-female groups/multi-male groups versus mixed samples of single-female/male and multi-female/male groups). For the reasons described in the introduction, we assumed that skew proxies that are calculated from mixed samples likely overestimate the reproductive skew.

Quantitatively, we indicated the combined sample size based on which we classified each species (i.e. the total number of groups and/or group-years of all proxies used to classify whether the species exhibits extreme reproductive skew or not). For species with conflicting evidence regarding the extent of reproductive skew (see §2(i)), we only considered the studies used for our final classification of the species. Samples that were used to calculate both types of proxies were only considered once. The sample size of the parentage-skew proxy was the number of groups that were used to calculate the proxy (though, when the number of groups was not clearly indicated by the authors, we used the number of group-years). The sample size of the skew-of-groups proxy was the number of groups or group-years from which we calculated the proxy (see details per study in electronic supplementary material, table S1).

## 3. Results

### (a) Mammals

For the 41 mammal species examined, we found 141 studies with relevant data on maternity and/or the care system (range: 0–9 studies per species; median: 3 studies per species with more than 0 studies). Eleven of these 41 species were excluded from our classification. Three of these species were excluded due to a lack of evidence of alloparental care, and eight species were excluded because we could not find data on maternity in wild populations or confirm the occurrence of multi-female groups (figure 3; electronic supplementary material, table S2). The datasets that originally classified these 11 species as cooperative breeders did not include supporting references for their classification (*n* = 4 species) or we were unable to verify the cited information in the references provided (*n* = 7 species).

#### (i) Extreme female reproductive skew in mammals

For the remaining 30 mammal species, we found evidence for alloparental care and extreme female reproductive skew in 11 species (figure 3; electronic supplementary material, table S2). However, only two of these 11 species were classified based on a relatively substantial combined sample size (i.e. ≥15 groups or ≥30 group-years) of multi-female groups (table 1; electronic supplementary material, table S2).

**Table 1.** Certainty of the classification of mammal species as exhibiting extreme female reproductive skew or not. The number of species according to the type of sample and combined sample size based on which we classified each species (*n* = 30 species; see electronic supplementary material, table S2 for details).

combined sample size:	type of sample	
	a mixed sample of groups with a single sexually mature female and groups with multiple sexually mature females	only groups with multiple sexually mature females
<15 groups or <30 group-years	2	14
≥15 groups or ≥30 group-years	5	9

### (b) Birds

For the 37 bird species examined, we found a total of 109 studies with relevant data on parentage and/or care system (range: 1–6 studies per species, median: 3 studies per species). Eight and six out of these 37 and 36 bird species were excluded from our classification of female/male reproductive skew, respectively. Six and three of the excluded species were excluded as not relevant to the question of reproductive skew in females/males, respectively (mostly due to a lack of evidence of alloparental care by sexually mature female/male alloparents; see figures 4 and 5; electronic supplementary material, table S2 for details). Additionally, respectively two and three of the excluded species were excluded because we could not find data on maternity/paternity in wild populations (figures 4 and 5; electronic supplementary material, table S2) and we were unable to verify the information cited by the datasets that classified these species as cooperative breeders.

#### (i) Extreme female reproductive skew in birds

For the remaining 29 bird species in our female dataset, we found evidence for alloparental care and extreme female reproductive skew in 12 bird species (including splendid fairy-wrens, which exhibited extreme reproductive skew in only one out of two populations examined; figure 4; electronic supplementary material, table S2). However, only two of these 12 species were classified based on a relatively substantial combined sample size (i.e. ≥15 groups or ≥30 group-years) of multi-female groups (table 2; electronic supplementary material, table S2).

**Table 2.** Certainty of the classification of bird species as exhibiting extreme female reproductive skew or not. The number of species according to the type of sample and combined sample size on which we based each species' classification (*n* = 29 species, although the table includes 30 entries as splendid fairy-wren was considered twice according to the two different populations with different degrees of reproductive skew; see electronic supplementary material, table S2 for details).

combined sample size:	type of sample	
	a mixed sample of groups with a single sexually mature female and groups with multiple sexually mature females	only groups with multiple sexually mature females
<15 groups or <30 group-years	3	10
≥15 groups or ≥30 group-years	6	11

#### (ii) Extreme male reproductive skew in birds

For the remaining 30 bird species in the male dataset, we found evidence of extreme male reproductive skew and alloparental care in eight species (including white-winged chough for which extreme reproductive skew depends on whether the group is newly formed or socially established; figure 5; electronic supplementary material, table S2). However, only one of these eight species was classified based on a relatively substantial combined sample size (i.e. ≥15 groups or ≥30 group-years) of multi-male groups (table 3; electronic supplementary material, table S2).

**Table 3.** Certainty of the classification of bird species as exhibiting extreme male reproductive skew or not. The number of species according to the type of sample and combined sample size on which we based each species' classification (*n* = 30 species; see electronic supplementary material, table S2 for details).

combined sample size:	type of sample	
	a mixed sample of groups with a single sexually mature male and groups with multiple sexually mature males	only groups with multiple sexually mature males
<15 groups or <30 group-years	1	13
≥15 groups or ≥30 group-years	5	11

## 4. Discussion

Our study revisits the restrictive definition of cooperative breeding, which assumes a quantitative difference between the reproductive skew of communally and cooperatively breeding species [22] (figure 1). After accounting for biases in how reproductive skew was assessed in previous datasets (figure 2), we report two main findings. First, the degree of reproductive sharing among cooperative and so-called communal breeding species largely overlaps (figure 1). Second, depending on the rigour of the data, a total of only 2–11 mammal and 2–12 bird species can be qualified as cooperative breeders according to the restrictive definition of cooperative breeding (electronic supplementary material, table S2).

### (a) Ignoring group composition when measuring proxies of reproductive skew results in logical and empirical faults

(1) At the logical level, including single-female and/or single-male groups in datasets may undermine the rationale of studies examining the causes or consequences of reproductive skew. This is because the restrictive definition of cooperative breeding requires alloparental care to be provided by individuals that could reproduce but do not actually do so [15,32]. Clearly, this combination of alloparental care by non-reproducing female/male helpers is absent in single-female/single-male groups [16,25].

For example, Raihani & Clutton-Brock [21] tested whether viviparity (i.e. the maintenance of developing embryos inside the female's reproductive tract) reduces the prevalence of reproductive suppression of subordinate females. To this end, the prevalence of female reproductive monopolization in mammals (i.e. viviparous species) versus birds (i.e. oviparous species in which embryos develop outside the female body) was compared. However, regardless of whether the species is viviparous or oviparous, in single-female groups, there are no female helpers whose reproduction could be suppressed by the ‘dominant’ females. Considering single-female groups in such an analysis is thus likely to obscure the true relationship between reproductive suppression, viviparity and oviparity.

(2) At the empirical level, including single-female or single-male groups in the calculation of reproductive skew proxies inflates the reproductive share of dominant females/males (figures 1–5). Across species, this inflation is expected to be positively correlated with the proportion of groups without alloparents. Specifically, populations with a high proportion of single-female/male groups (see review in [12]), will be classified as exhibiting extreme reproductive skew even if reproduction is shared within the few multi-female and/or multi-male groups existing in the population (see examples in figures 2–5).

(3) We note that also the reproductive skew proxies reported here likely overestimate the reproduction share of dominant adults for two reasons. First, in the absence of more accurate data for some species, we calculated skew proxies from mixed samples that may include single-female groups/single-male groups (tables 1–3). Second, group members may not reproduce simultaneously. Short-term studies that only assessed the number of reproductive females/males during a short period (e.g. by counting pregnant females [56,57]) may overlook asynchronous reproductive sharing [39,58]. The skew-of-groups proxy is therefore prone to overestimation especially when it is based on short-term studies that do not also assess the dominance rank of breeders [37].

(4) We addressed additional causes for overestimating the reproductive share of dominant individuals (figure 2 and §2c) which should be avoided in future studies (see box 1). That is, (i) the inclusion of extra-group parentage [37], (ii) the inclusion of broods/litters with a single sampled offspring in estimating the skew-of-groups proxy when the sample includes one breeding attempt per group (as shared parentage cannot be detected/occur in these broods/litters [26]), and (iii) focusing on a single breeding attempt per group (as reproduction may be shared across breeding attempts [37,59]).

Box 1. **Guidelines for reporting data on parentage in cooperative breeding species (amended from Brouwer & Griffith [37])**

A. Contextual information Indicate:

1. the basic unit in the social system of the study species (in most species this will be a social group consisting of two or more breeding individuals and alloparents; in species living in multi-level societies, such as white-fronted bee-eaters, *Merops bullockoides* [50,51], and superb starlings, *Lamprotornis superbus* [48], it may be a larger social group, e.g. a ‘clan’, consisting of multiple sub-units);
2. the location of the study population (latitude and longitude) and previous parentage studies of that population;
3. the sampling period;
4. whether and how the dominance ranks and/or social pairing within the basic social unit were inferred;
5. the reproductive stage at which parentage data were collected (pregnancy/dependent offspring/independent offspring);
6. whether the focal animals were subject to human-induced manipulation that may alter reproductive sharing within social units (e.g. experimental, captive or re-introduced animals).

B. General sample size Indicate the:

1. number of basic social units sampled;
2. number of broods/litters sampled;
3. number of offspring sampled.

C. Sub-samples

For each offspring's maternity and paternity data entry (and for each brood/litter as a whole) report the following data to enable the calculation of reproductive sharing under different social compositions (see also figure 2):

1. Were all sexually mature members in the basic social unit sampled (i.e. all potential parents)? (yes/no)
2. Was the offspring produced by a male outside of the offspring's social group? (i.e. extra-group parentage) similar data for females.
3. What is the social status of the offspring's father within the social group? (dominant/subordinate) similar data for the mother.
4. Was the offspring the only one sampled in its brood/litter? (i.e. to verify whether shared parentage could be detected/occurring in this brood/litter) (yes/no).
5. Did the offspring's basic social unit include at least one male alloparent (reproductive or non-reproductive)? (yes/no) similar data for females.
6. Did the offspring's basic social unit include multiple sexually mature males? (yes/no) similar data for females.
7. Did the offspring's basic social unit include multiple sexually mature males that were unrelated to a sexually mature female in their group? (i.e. social units with/without incest limitation) (yes/no) similar data for females.
8. Did the composition of sexually mature members in the offspring's basic social unit remain the same as the previous breeding attempt? (i.e. to examine reproductive sharing across reproductive attempts [37,59]) (yes/no).

Below is a hypothetical example of reporting parentage according to the above guidelines.

**Table:** 

the basic social unit of the species	a family unit with alloparents	a family unit with alloparents
study site location	04°40′S, 55°40′ E	04°40′S, 55°40′ E
sampling date	01.01.2023	01.01.2023
offspring ID	1234	4567
brood ID	100	200
basic social unit ID	‘yellow’	‘green’
the reproductive stage at sampling	dependent offspring	dependent offspring
was the group subjected to a human-induced manipulation that may alter reproductive sharing?	no	yes (experiment)
were all sexually mature members in the basic social unit sampled?	yes	yes
was the offspring the only one sampled in its broods/litters?	no	yes
father ID	1111	3333
father social status	dominant	dominant
was the offspring produced by a male outside of the offspring's social group? (i.e. extra-group parentage)	no	yes
mother ID	2222	4444
mother social status	dominant	subordinate
was the offspring produced by a female outside of the offspring's social group? (i.e. extra-group parentage)	no	no
did the offspring's basic social unit include at least one male alloparent (reproductive or non-reproductive)?	yes	yes
did the offspring's basic social unit include at least one female alloparent (reproductive or non-reproductive)?	no	yes
did the offspring's basic social unit include multiple sexually mature males?	yes	yes
did the offspring's basic social unit include multiple sexually mature females?	no	yes
did the offspring's basic social unit include multiple sexually mature males that were unrelated to a sexually mature female in their group?	no	yes
did the offspring's basic social unit include multiple sexually mature females that were unrelated to a sexually mature male in their group?	no	no
did the composition of sexually mature members in the offspring's basic social unit remain the same as the previous breeding attempt?	yes	no

### (b) Inflation of within-group reproductive skew inflates the prevalence of species exhibiting extreme reproductive skew

(5) False-positive classifications of species as exhibiting extreme reproductive skew lead to overestimating the number of species qualified as cooperative breeders by restrictive definitions. Indeed, after partly accounting for biases in estimating reproductive skew, we shorten the lists of species with evidence of alloparental care and extreme reproductive skew by at least 73% for mammals and 68% for birds, depending on the criteria of rigorous data (see also [45]).

(6) This significant change in the number and identity of species that can be classified as cooperative breeders according to restrictive definitions has two important consequences. First, there is a need to revisit comparative studies that used previous datasets [see also 17,37,60] and the updated datasets provided here and elsewhere [12,37] facilitate these revisions. Second, the very small number of cooperative breeding species according to restrictive definitions reduces the ability to carry out statistically meaningful analyses to assess the causes and consequences of alloparental care [38]. It therefore also questions the practicality of the restrictive definitions of cooperative breeding.

(7) Our results demonstrate a wide continuum of within-group reproductive sharing even within the group of species that were claimed to exhibit extreme reproductive skew (figure 1). This finding questions the underlying biological assumption of restrictive definitions regarding a quantitative gap between so-called communal breeding versus cooperative breeding species [22]. It also provides the first systematic evidence supporting Sherman *et al.* [38] and Riehl's [34,61] proposal that within-group reproductive sharing among cooperative-breeding species is better seen as a continuum, in which the few species with an extreme reproductive skew represent one end rather than a qualitatively different category.

We therefore join many other behavioural ecologists [35,62–68] and evolutionary anthropologists [69,70] in defining cooperative breeding as the provision of systematic alloparental care regardless of the reproductive status of alloparents. The extent of within-group reproductive sharing can then be examined as one of the continuous characteristics of cooperative breeding species [12,34,38]. This inclusive approach maintains the distinction between mating and caring systems [2] and the primary interest in cooperative breeding as a caring system with alloparental care [1,71].

### (c) Curating datasets on cooperative breeding species

(8) This work seeks to facilitate the constant improvement of datasets on cooperative breeding species. We therefore provide estimations for the strength of the evidence supporting each species' classification (electronic supplementary material, table S2). These estimations should direct research efforts towards species that require further study and facilitate statistical control for data uncertainty. The latter is particularly important due to the high proportion of species whose classification is based on mixed samples or limited sample sizes (tables 1–3). Following Brouwer & Griffith [37], we also provide guidelines for reporting parentage data in cooperative breeding species (box 1 and figure 2).

(9) Our study demonstrates that a comprehensive review of the primary literature and fine filtering of those social groups that are relevant to the specific question addressed in a given study are crucial. These laborious reviewing tasks require qualified readers and extensive time that should not be underestimated [37]. We join biologists [17,72,73] and statisticians [74,75] in suggesting that, in the absence of these resources, meta-analyses should be based on smaller and more accurate datasets, rather than larger but less accurate ones. Small high-quality datasets can in the future be merged into comprehensive ones, thereby ensuring a better understanding of cooperative breeding in the longer perspective. Following this rationale, we provide a comprehensive account of within-group reproductive sharing in 63 cooperative breeding mammal and bird species, hoping it will be joined by comprehensive reviews of additional cooperative breeding species in the future.

## Data Availability

All data presented in this study are included in electronic supplementary material, tables S1–S2 [76].
